# Host Factors Involved in the Intracellular Movement of *Bamboo mosaic virus*

**DOI:** 10.3389/fmicb.2017.00759

**Published:** 2017-04-25

**Authors:** Chi-Ping Cheng

**Affiliations:** Department of Life Sciences, Tzu Chi UniversityHualien, Taiwan

**Keywords:** *Bamboo mosaic virus*, intracellular movement, vesicle trafficking, endomembrane system, host factors

## Abstract

Viruses move intracellularly to their replication compartments, and the newly synthesized viral complexes are transported to neighboring cells through hijacking of the host endomembrane systems. During these processes, numerous interactions occur among viral proteins, host proteins, and the cytoskeleton system. This review mainly focuses on the plant endomembrane network, which may be utilized by *Bamboo mosaic virus* (BaMV) to move to its replication compartment, and summarizes the host factors that may be directly involved in delivering BaMV cargoes during intracellular movement. Accumulating evidence indicates that plant endomembrane systems are highly similar but exhibit significant variations from those of other eukaryotic cells. Several *Nicotiana benthamiana* host proteins have recently been identified to participate in the intracellular movement of BaMV. Chloroplast phosphoglycerate kinase, a host protein transported to chloroplasts, binds to BaMV RNAs and facilitates BaMV replication. NbRABG3f is a small GTPase that plays an essential role in vesicle transportation and is also involved in BaMV replication. These two host proteins may deliver BaMV to the replication compartment. Rab GTPase activation protein 1, which switches Rab GTPase to the inactive conformation, participates in the cell-to-cell movement of BaMV, possibly by trafficking BaMV cargo to neighboring cells after replication. By analyzing the host factors involved in the intracellular movement of BaMV and the current knowledge of plant endomembrane systems, a tentative model for BaMV transport to its replication site within plant cells is proposed.

## Introduction

Membrane trafficking delivers materials between the endomembrane system and the plasma membrane and therefore plays an essential role in cell survival and development (Cheung and de Vries, 2008). Many animal microbes that reproduce intracellularly, including viruses and bacteria, have been shown to utilize host endomembrane trafficking and the autophagy system for intracellular transport within the host cells (Cossart and Helenius, 2014; Yamauchi and Greber, 2016), whereas other microbes alter the degradation pathway used by the host cells to destroy the intruding pathogens (Miller and Krijnse-Locker, 2008; Mudhakir and Harashima, 2009; Yamauchi and Greber, 2016). Rab proteins are small GTPases involved in membrane trafficking of cells, specifically in vesicle formation and fusion. Recent studies of *Salmonella enterica* Typhimurium, an animal pathogenic bacterium, have identified several bacterial effectors that target various Rab proteins for replication within the host cells (Spano et al., 2011, 2016; McGourty et al., 2012; D’Costa et al., 2015).

Many plant viruses induce membrane remodeling after infecting their host cells. Studies focusing on the modification of endomembrane systems induced by plant viruses have substantially improved our understanding of the intracellular movement of plant viruses. Membrane targeting or recruitment by viral proteins in the viral replication compartment is often the cause of endomembrane remodeling, as has been demonstrated for many viruses (Wei et al., 2010, 2013; Hyodo et al., 2014; Xu and Nagy, 2014). *Grapevine fanleaf nepovirus* (GFLV) infection induces structural changes in the endoplasmic reticulum (ER) membranes to allow virus replication in the ER-derived membrane. Although host factors have not been identified, inhibitor treatment has demonstrated that vesicle transport between Golgi and ER is essential for GFLV replication (Ritzenthaler et al., 2002; Fuchs et al., 2017). A study on plant potyviruses revealed that the 6k2 viral protein of *Turnip mosaic virus* (TuMV) induces the formation of vesicles derived from ER and targets them to the chloroplasts for replication (Wei et al., 2010). *Melon necrotic spot virus* (MNSV) has been shown to replicate in mitochondria, which is significantly altered by the virus-encoded p29 protein targeting the mitochondria membrane (Mochizuki et al., 2009; Gomez-Aix et al., 2015). ER disorder has also been observed, and the p7B movement protein of MNSV is localized to ER, Golgi, actin filaments, and plasmodesmata. Disruption of the transport between ER and Golgi results in the accumulation of p7B within the ER. Therefore, the ER-to-Golgi secretory pathway could be involved in the intra- and intercellular movement of MNSV (Genoves et al., 2010).

In addition to the viral encoded proteins, host factors are also involved in the membrane remodeling process. Using yeast as a model system and by performing further testing in host plants, several studies on *Tomato bushy stunt virus* (TBSV) have demonstrated that host factors are responsible for delivering components to remodel the membrane-associated compartment for viral replication (Barajas et al., 2014; Xu and Nagy, 2016). Heat shock protein 70 is associated with the replication complex of TBSV and facilitates the insertion of viral replication proteins into the yeast membrane (Wang et al., 2009). The GTP-bound active form of Rab5-positive endosome is hijacked by TBSV for enrichment of phosphatidylethanolamine at the replication site (Xu and Nagy, 2016). A host SNARE protein, Syp71, mediates the fusion between the TuMV-induced vesicles and chloroplasts, which is required for TuMV infection (Wei et al., 2013). In yeast, membrane-shaping reticulon homology domain proteins are crucial for the formation of the replication compartment induced by the *Brome mosaic virus* (BMV) 1a protein (Diaz et al., 2010). These findings indicate that membrane trafficking and targeting are essential processes for plant virus replication.

Several pathways of intracellular movement have been proposed for animal viruses after they enter the host cells (Mudhakir and Harashima, 2009). By contrast, the trafficking pathways for the intracellular movement of plant viruses to their replication sites within the host cells remain largely unknown. Particularly, the endomembrane trafficking systems in plants seem to be more complicated and have not been completely revealed (Saito and Ueda, 2009; Uemura, 2016). Moreover, studies of the intracellular movement of plant viruses have mostly focused on cell-to-cell movement through the plasmodesmata; these intracellular movement pathways of plant viruses have been reviewed in several articles (Park et al., 2014; Heinlein, 2015; Liou et al., 2015). Host factors participating in membrane trafficking or protein targeting may play roles in delivering BaMV or its cargoes to the replication sites. Based on the current knowledge of intracellular trafficking pathways in plants, a model for the intracellular movement of BaMV to its replication compartment is proposed. After replication, plant viruses travel intracellularly to reach plasmodesmata for cell-to-cell movement. A vesicle trafficking-related host protein participates in BaMV cell-to-cell movement; its potential role in BaMV intracellular movement is also discussed in this review.

## Possible Replication Compartments for BaMV

Virus infection commonly induces the formation of dynamic membrane-associated structures that are associated to the virus replication and movement (Grangeon et al., 2012; Heinlein, 2015). Chloroplasts are one of the types of compartments suitable for plant virus replication (Wei et al., 2010; Zhao et al., 2016). Using BaMV 3′ RNA as a probe for *in situ* hybridization through electronic microscopy, BaMV viral RNAs were detected within several organelles of green bamboo leaf cells, including chloroplasts, mitochondria, and nuclei (Lin et al., 1993). Phage MS2 coat protein can specifically bind to its own RNA sequence, and viral genomic RNA engineered to contain the MS2 sequence can be traced within the cells through the binding of GFP-fused MS2 coat protein (Zhang and Simon, 2003). Recently, through confocal microscopy, BaMV viral RNA expressing the phage MS2 coat protein binding sequence was found to localize within chloroplasts. Furthermore, negative-strand BaMV RNA was found in the isolated chloroplasts, demonstrating that chloroplasts may be among the BaMV replication compartments within the host cells (Cheng et al., 2013a).

In contrast, *Potato virus X* (PVX), another potexvirus, has been found to replicate in the ER membrane (Doronin and Hemenway, 1996; Park et al., 2014); in addition, TGB2/3 has recently been shown to remodel the ER membrane at plasmodesmata, where PVX coupled the replication and movement to the neighboring cells (Tilsner et al., 2013).

## Host Factors Probably Involved in the Intracellular Targeting of BaMV to the Replication Compartment

After entering the host cells, similar to other viruses, BaMV must travel to its replication site intracellularly. Recently, several host factors involved in BaMV replication have been identified through copurification with the BaMV replicase complex (Cheng et al., 2009; Prasanth et al., 2011; Huang et al., 2012; Lee et al., 2015), binding to the viral RNAs (Lin et al., 2007; Cheng et al., 2013a), and cDNA-amplified fragment length polymorphism (cDNA-AFLP) analysis (Chen et al., 2013; Huang et al., 2013, 2016). Among these host factors, three are possibly involved in the transportation of BaMV or its related cargoes within host cells. Chloroplast phosphoglycerate kinase (chl-PGK) (Lin et al., 2007; Cheng et al., 2013a) and NbRABG3f (Huang et al., 2016) are related to BaMV replication, whereas NbRabGAP1 is essential for the intercellular movement of BaMV (Huang et al., 2013).

In *Nicotiana benthamiana*, chl-PGK, a nuclear-encoded chloroplast protein, was found to bind to the 3′ untranslated region (3′ UTR) of BaMV genomic RNAs (Lin et al., 2007). Knocking down the expression or mistargeting of chl-PGK reduced BaMV accumulation in *N. benthamiana* protoplasts, indicating that chl-PGK participates in BaMV replication (Cheng et al., 2013a). Redirecting a BaMV RNA binding protein, EF1a, to chloroplasts rescued the reduction of BaMV caused by the knock-down of chl-PGK. These results demonstrate that chl-PGK likely assists in the targeting of BaMV to the chloroplasts for replication (Cheng et al., 2013a). Recently, a chl-PGK from *Arabidopsis thaliana* was identified as a requirement for efficient *Watermelon mosaic virus* (WMV) (Ouibrahim et al., 2014) and *Plum pox virus* (Poque et al., 2015) infection. Although the replication compartment of WMV is unknown, the 6K protein of other potyviruses induces the formation of mobile vesicles and transports the viruses from the ER to the chloroplast membrane for viral replication (Wei et al., 2010). Therefore, chl-PGK may be involved in the intracellular transport of different plant viruses to the chloroplasts for replication.

A recent study identified that NbRABG3f, a Rab small GTPase, is involved in BaMV replication (Huang et al., 2016). Rab small GTPases are involved in vesicle trafficking within cells. Rab proteins alternate between a GTP-bound active form and GDP-bound inactive form, and this switching is accelerated by their regulatory proteins (Cherfils and Zeghouf, 2013). GTP-bound Rab proteins bud from the donor compartment and fuse with the acceptor compartment, where GTP is hydrolyzed by GTPase-activating proteins (GAPs) (Cherfils and Zeghouf, 2013). Based on sequence comparison, NbRABG3f is homologous to animal cell Rab7 protein and to *A. thaliana* RabG3f (Huang et al., 2016). cDNA-AFLP analysis revealed that NbRABG3f expression was upregulated after BaMV inoculation (Cheng et al., 2010). The GDP-bound form of Rab GTPase can be used to trace the donor compartment of Rab proteins (Sieczkarski and Whittaker, 2002); thus, confocal microscopy revealed that the GDP-bound form mutant of NbRABG3f was localized to the Golgi compartment (Huang et al., 2016). In plant endomembrane trafficking systems, endocytotic materials are transferred to the *trans*-Golgi network for further sorting (Zhuang et al., 2015). As a plant defense mechanism pathway, intruding pathogens are likely delivered to the multi-vesicular body (MVE)/prevacuolar compartment (PVC), or autophagosome and then delivered to the vacuoles for degradation (Teh and Hofius, 2014). Successful pathogen infection results from a redirection of the pathway to their replication compartments rather than the degradation pathway (Patarroyo et al., 2012; Dong and Levine, 2013). According to the finding that NbRABG3f is derived from the Golgi compartment (Huang et al., 2016) and that chloroplasts are likely to be the replication sites of BaMV (Lin et al., 1993; Cheng et al., 2013a), BaMV may utilize NbRABG3f or NbRABG3f-associated vesicles for transport to chloroplasts. However, the acceptor membrane of NbRABG3f has not yet been identified. Knowledge of the destination of NbRABG3f in the endomembrane systems will verify whether BaMV hijacks NbRABG3f and redirects its route or instead utilizes NbRABG3f-associated vesicle and follows its pathway. Further investigation of the interaction of BaMV with NbRABG3f and other Rab proteins and further evaluation of the colocalization of BaMV and various marker proteins involved in endomembrane trafficking will reveal the intracellular transportation pathway for BaMV.

## Possible Model for BaMV Intracellular Movement to its Replication Compartment

In uninfected *N. benthamiana* cells, NbRABG3f and its associated vesicles are generated by the Golgi compartment (Huang et al., 2016). The destination of NbRABG3f is currently unknown. According to a putative model proposed for *Arabidopsis*, the endocytosed materials can be (1) delivered to the MVE/PVC, or autophagosome and then transported to the vacuoles for degradation, or (2) recycled into the plasma membrane (Uemura, 2016; Vukasinovic and Zarsky, 2016). A previous study demonstrated that *Arabidopsis* AtRABG3f mediates transport from PVC to the vacuole (Cui et al., 2014). Although the donor membrane of NbRABG3f is different from that of AtRABG3f, possibly because of variation in the C-terminal sequence, based on the fact that NbRABG3f is highly homologous to AtRABG3f (Huang et al., 2016), NbRABG3f likely participates in the transport of vesicles to other endomembrane systems rather than in their delivery to the recycling pathway.

**FIGURE 1:**
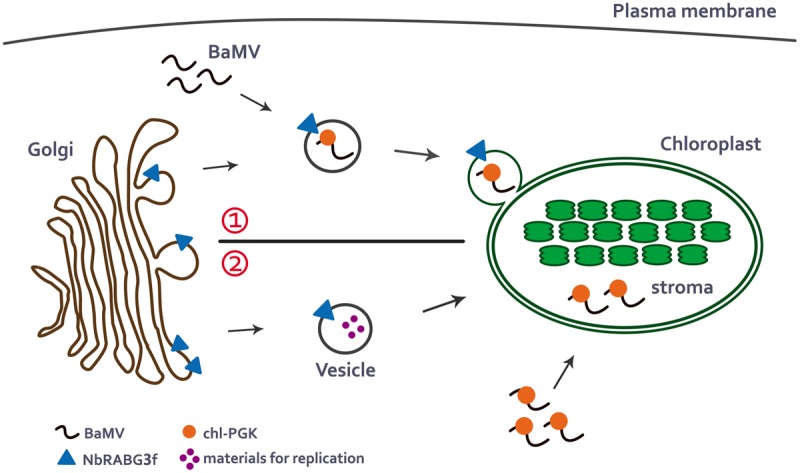
**A possible model for BaMV intracellular movement to the chloroplasts.** In the normal membrane-trafficking pathway, NbRABG3f-associated vesicles bud from Golgi compartment. There are two possible pathways that NbRABG3f and chl-PGK may participate in delivering of BaMV cargoes to the chloroplasts. (1) When BaMV infects the cells, it may hijack the NbRABG3f-associated vesicles and redirect the vesicles to the chloroplast which is one of its replication compartments. During the transportation to the chloroplasts, chl-PGK is recruited to the BaMV complex and targets BaMV complex to the chloroplasts stroma **(Upper)**. (2) Alternatively, NbRABG3f-associated vesicles may deliver materials required for BaMV replication to the chloroplasts and chl-PGK binds to BaMV viral RNA and targets it to the chloroplast stroma for replication **(Lower)**.

According to current knowledge of the intracellular trafficking pathways in plants and studies of NbRABG3f and chl-PGK, BaMV may utilize NbRABG3f-associated vesicles for vesicle trafficking. During vesicle trafficking, chl-PGK is recruited and assists in the targeting of the BaMV complex to the chloroplasts, which are one of the types of BaMV replication compartments (Cheng et al., 2013a) (**Figure 1**, upper). By contrast, similar to the role of Rab5 protein in TBSV replication (Xu and Nagy, 2016), NbRABG3f may deliver the materials required for BaMV replication to the chloroplasts, and chl-PGK may bind BaMV RNA and direct the BaMV complex to the chloroplast for replication (**Figure 1**, lower).

Although the transportation pathway for chloroplast-targeting proteins to the chloroplast is not completely clear, to date, several pathways have been proposed for proteins transported to the chloroplasts after translation (Radhamony and Theg, 2006; Shi and Theg, 2013). Most chloroplast proteins contain an N-terminal cleavable transit peptide that mediates the targeting of the protein to the chloroplasts (Shi and Theg, 2013). *N. benthamiana* chl-PGK also contains a putative N-terminal transit peptide (Cheng et al., 2013a), indicating that it can be transported from the ER to the chloroplast stroma, and that such transport is directed by the N-terminal transit peptide. Whether chl-PGK is recruited to the NbRABG3f-associated vesicle or these two host proteins are involved in separate steps of BaMV transportation is an interesting question that remains to be explored. Future studies should investigate whether chl-PGK interacts with NbRABG3f or NbRABG3f-associated vesicles during the transportation process.

## A Host Factor Possibly Involved in BaMV Vesicle Trafficking to the Plasmodesmata for Intercellular Movement

After the replication of plant viruses, virion or virus ribonucleoprotein (vRNP) facilitates their intercellular movement through the plasmodesmata. Unlike PVX, which moves intercellularly as vRNP, BaMV is likely to move as a virion associated with TGBp2 and TGBp3-based ER membrane (Chou et al., 2013). Several BaMV viral proteins and *N. benthamiana* host factors have been demonstrated to facilitate the intercellular movement, but not the replication, of BaMV (Cheng et al., 2013b; Huang et al., 2013; Hung et al., 2014). A recent, thorough review of the intercellular movement of BaMV hypothesizes the possible roles of the viral and host proteins in the intercellular movement of BaMV (Liou et al., 2015). Among the host proteins involved in the intercellular movement of BaMV, NbRabGAP1, a Rab-GTPase activation protein, has been identified through cDNA-AFLP analysis, and its expression is upregulated after BaMV inoculation (Huang et al., 2013). RabGAP contains the TBC (Tre2/Bub2/Cdc16) catalytic domain that can promote GTP hydrolysis and thus inactivates Rab proteins (Frasa et al., 2012). Rab proteins participate in the regulation of vesicle formation and trafficking in the endomembrane system. In the study of NbRabGAP1, low NbRabGAP1 expression reduced BaMV accumulation in *N. benthamiana* leaves, but not in protoplasts, whereas overexpression of NbRabGAP1 exerted the opposite effect. Based on these results, it is hypothesized that NbRabGAP1 is involved in the delivery of the BaMV/BaMV-related cargo from the virus replication complex to neighboring cells (Huang et al., 2013; Liou et al., 2015). An attempt to examine the interaction between NbRabGAP1 and NbRABG3f was unsuccessful (unpublished data). This outcome was expected because NbRabG3f participates in BaMV replication, whereas NbRabGAP1 assists in the intercellular movement of BaMV. Similar to PVX, the BaMV movement proteins TGBp2 and TGBp3 are ER-targeting membrane proteins (Hsu et al., 2008). The BaMV infectious complex has been proposed to move from perinuclear ER-derived membrane-bound bodies (MBB) to the plasmodesmata (Wu et al., 2011; Liou et al., 2015). After BaMV replication, NbRabGAP1 possibly participates in the delivery of BaMV cargoes with an unknown Rab protein toward the plasmodesmata through ER and post-ER secretory pathways. Alternatively, NbRabGAP1 may recycle the BaMV movement proteins from plasmodesmata to assist in the next round of BaMV transportation (Huang et al., 2013). However, the connection between chloroplasts and the MBB requires further investigation to unveil the intracellular route after BaMV replication.

Studies in PVX have indicated that its TGB2/3 movement proteins induce ER-derived granular vesicles, which are essential for PVX cell-to-cell movement (Ju et al., 2007); therefore, the PVX movement complex may be transported through these vesicles to plasmodesmata (Verchot-Lubicz et al., 2010). Accordingly, as yet unidentified host factors may be present that function as Rab or RabGAP proteins to facilitate PVX movement.

The kinases CK2 and NbSTKL are other host factors also involved in the cell-to-cell movement of BaMV (Cheng et al., 2013b; Hung et al., 2014). Knowledge on host factors that affect the intercellular movement of potexviruses have been reviewed recently (Park et al., 2014; Liou et al., 2015). Their functions in assisting BaMV or other potexvirus movement have also been discussed in a recent review, which reported that they might not directly participate in the transportation of BaMV cargoes (Liou et al., 2015). Therefore, they are not included in this review.

## Summary

In this review, I summarized the host factors that participate in membrane trafficking and a specific chloroplast targeting protein that may participate in BaMV transportation within the cells. A possible model was proposed to demonstrate how BaMV is delivered to its replication compartment. One of the host factors, NbRABG3f, is a small GTPase that mediates vesicle trafficking and is derived from the Golgi compartment. Both GTPase activity and membrane-targeting ability are essential for BaMV replication. Another host protein, chl-PGK, is a chloroplast-targeting protein, and its targeting ability is required for BaMV replication. Therefore, NbRABG3f and chl-PGK may play roles in delivering BaMV and its related complex to chloroplasts, which are a type of BaMV replication compartment. After completing replication, BaMV cargoes are delivered to the plasmodesmata for intercellular movement. NbRabGAP1, a Rab-associated protein, is not involved in BaMV replication but participates in its intercellular movement. Accordingly, NbRabGAP1 may be involved in the intracellular transport of the BaMV complex to the plasmodesmata after BaMV replication. Additional studies on dissecting the co-localization or interaction between BaMV and other vesicle proteins are required to clarify the intracellular movement pathway for BaMV.

## Author Contributions

C-PC wrote and edited the paper.

## Conflict of Interest Statement

The author declares that the research was conducted in the absence of any commercial or financial relationships that could be construed as a potential conflict of interest.
